# Rash with DERMABOND PRINEO Skin Closure System Use in Bilateral Reduction Mammoplasty: A Case Series

**DOI:** 10.1155/2015/642595

**Published:** 2015-04-01

**Authors:** R. W. Knackstedt, J. A. Dixon, P. J. O'Neill, F. A. Herrera

**Affiliations:** MUSC Division of Plastic Surgery, 96 Jonathan Lucas Street, CSB 404, MSC 613, Charleston, SC 29425, USA

## Abstract

*Background*. Bilateral reduction mammoplasty is a common plastic surgery procedure that can be complicated by unfavorable scar formation along incision sites. Surgical adhesives can be utilized as an alternative or as an adjunct to conventional suture closures to help achieve good wound tension and provide an adequate barrier with excellent cosmesis. The recently introduced DERMABOND PRINEO Skin Closure System Skin Closure System combines the skin adhesive 2-octyl cyanoacrylate with a self-adhering polyester-based mesh. Proposed benefits of wound closure with DERMABOND PRINEO Skin Closure System, used with or without sutures, include its watertight seal, easy removal, microbial barrier, even distribution of tension, and reduction in wound closure time. Although allergic reactions to 2-octyl cyanoacrylate have been reported, few allergic reactions to DERMABOND PRINEO Skin Closure System have been noted in the literature. This case series describes three patients who experienced an allergic reaction to DERMABOND PRINEO Skin Closure System after undergoing elective bilateral reduction mammoplasties at our institution to further explore this topic. *Methods*. Retrospective chart review of bilateral reduction mammoplasty patients who received DERMABOND PRINEO Skin Closure System dressing at our institution was performed. *Results*. Three patients were identified as having a rash in reaction to DERMABOND PRINEO Skin Closure System after bilateral reduction mammoplasty. All three patients required systemic steroid treatment to resolve the rash. One patient was identified as having a prior adhesive reaction. *Conclusions*. DERMABOND PRINEO Skin Closure System has demonstrated its efficacy in optimizing scar healing and appearance. However, as we demonstrate these three allergic reactions to DERMABOND PRINEO Skin Closure System, caution must be utilized in its usage, namely, in patients with a prior adhesive allergy and in sites where moisture or friction may be apparent.

## 1. Background 

Reduction mammoplasty can be complicated by scar formation. Surgical adhesives can help achieve optimal wound tension and provide excellent cosmesis. DERMABOND PRINEO Skin Closure System combines 2-octyl cyanoacrylate with a self-adhering polyester-based mesh. Proposed benefits of DERMABOND PRINEO Skin Closure System include its watertight seal, easy removal, microbial barrier, even distribution of tension, and reduction in wound closure time [1–3].

Although allergic reactions to 2-octyl cyanoacrylate have been reported [4], few reactions to DERMABOND PRINEO Skin Closure System have been reported [1, 3, 5]. This series highlights three patients who experienced an allergic reaction to DERMABOND PRINEO Skin Closure System after undergoing elective bilateral reduction mammoplasties.

## 2. Case 1

**Figure 1:**
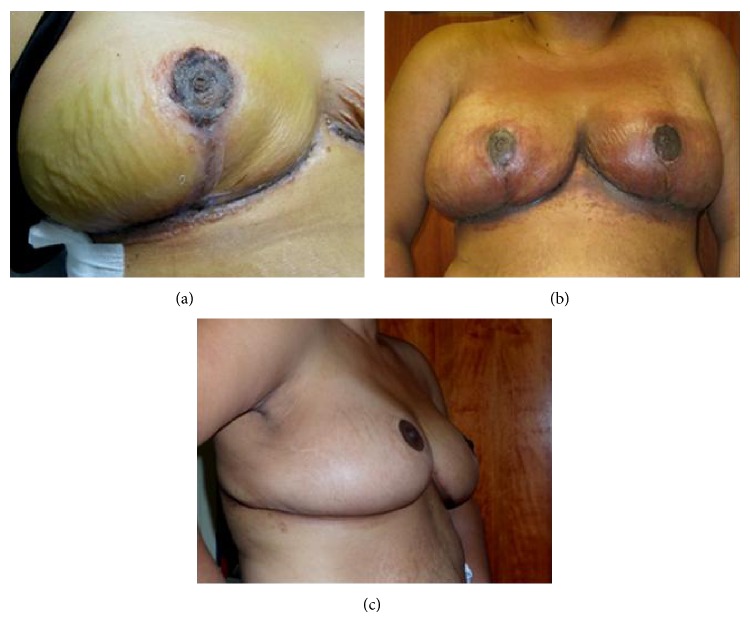
Progression of rash in patient. (a) Patient at initial follow-up postoperation day (POD) 6 with bilateral swelling, blisters, and erythema. (b) Patient at POD 11 after 5 days of steroid taper. Steroid taper was subsequently terminated due to gastrointestinal intolerance. Rash had spread bilaterally since initial presentation. (c) Resolution of rash at POD 19.

A 33-year-old Caucasian woman with a BMI of 30.5 and a documented penicillin allergy (Table 1) was prepped with chlorhexidine gluconate and the following sutures were utilized: silk for drains, deep polyglactin 910, subcuticular poliglecaprone 25, and poliglecaprone 25 for the nipple. Six days later, bilateral breast swelling, blisters, and eczematous, erythematous patches near the DERMABOND PRINEO Skin Closure System were noted. Diphenhydramine did not relieve the pruritus. An oral steroid taper pack and hydroxyzine were prescribed. Although the dermatitis was not consistent with a drug rash, the cephalexin was stopped due to the risk for cross reactivity. Steroid taper was discontinued after five days due to gastrointestinal intolerance. At this time, the rash had intensified and spread from chest to arms, neck, and face. All drains and residual DERMABOND PRINEO Skin Closure System were removed; the skin was washed with saline and covered with sterile mesh gauze impregnated with petrolatum 3% bismuth tribromophenate (Xeroform) and petrolatum ointment. A steroid taper was restarted and dermatology consult was made. Two days later, the rash had greatly improved. After six additional days, there was no trace of the rash (Figure 1).

## 3. Case 2

A 31-year-old Caucasian woman with a BMI of 24.2 with no history of allergic contact dermatitis or drug allergy (Table 1) was prepped with chlorhexidine gluconate and the following sutures were utilized: deep polyglactin 910, subcuticular poliglecaprone 25, polyglactin 910, and rapidly absorbing plain gut for the nipple. Five days later, there was no rash. Twenty-three days later, a pruritic, erythematous rash along the intramammary incision was noted. Diphenhydramine did not alleviate symptoms. The remaining DERMABOND PRINEO Skin Closure System was removed. The patient was given prednisone and scheduled with dermatology. One month later, the rash had resolved.

## 4. Case 3

A 38-year-old African American woman with a BMI of 28.13 and an allergy to codeine and iodine (Table 1) was prepped with chlorhexidine gluconate and the following sutures were utilized: deep polyglactin 910, deep poliglecaprone 25, and intracuticular poliglecaprone 25. The patient presented eight days later with numerous erythematous papules on the upper poles of the breasts that had presented that morning. Over-the-counter hydrocortisone cream had not alleviated symptoms. She was instructed to continue hydrocortisone. Ten days later, the rash had spread to her flexor forearms. She began a methylprednisone taper pack and the rash resolved within two days.

## 5. Discussion

DERMABOND PRINEO Skin Closure System combines 2-octyl cyanoacrylate with a self-adhering polyester-based mesh and is marketed as allowing for improved cosmesis with reductions in healing time and complications [1–3]. Studies have found that 2-octyl cyanoacrylate is a safe and effective option for skin closure [6–11]. A paucity of reports have focused on the potential side effects of DERMABOND PRINEO Skin Closure System [1, 3, 5] which utilizes this skin adhesive. This case report discusses allergic contact reactions to DERMABOND PRINEO Skin Closure System observed at our institution in patients undergoing reduction mammoplasties.

Few DERMABOND PRINEO Skin Closure System side effects have been reported in the literature. One study comparing sutures to DERMABOND PRINEO Skin Closure System in patients did not report any allergic reactions [3]. In a retrospective study, 4 of 224 patients (1.8%) experienced an allergic dermatitis [1]. However, these patients had prior DERMABOND PRINEO Skin Closure System exposure, suggesting sensitization, consistent with a type IV hypersensitivity or cutaneous allergic contact dermatitis [1]. None of our patients had previous DERMABOND PRINEO Skin Closure System exposure. A limitation of our study is that patients were not patch tested and we cannot rule out contact dermatitis. However, we would expect a greater patient population to demonstrate similar rashes if it were a contact dermatitis. As it has been suggested that DERMABOND PRINEO Skin Closure System not be used for patients with a known or suspected allergy or sensitivity to cyanoacrylate, formaldehyde, tapes, or adhesives [3], it would be interesting to determine if individuals with documented skin allergy are more prone to reactions. The likely allergen of DERMABOND PRINEO Skin Closure System, cyanoacrylate, has wide-spread surgical and nonsurgical uses. Thus, patients must be screened for all past allergic reactions. The patients in the prior study were managed via topical corticosteroids and 20% azelaic acid [1]. Our patients were primarily treated with oral steroids.

In our report, there were no observed wound infections. One study did find a nonstatistically significant increase in infections (2 versus 1 patient) in wounds closed with DERMABOND PRINEO Skin Closure System versus sutures. The authors suggested this could be due to occlusion by DERMABOND PRINEO Skin Closure System [3].

DERMABOND PRINEO Skin Closure System may be just as, if not more, desirable as sutures. Studies have shown that patients prefer DERMABOND PRINEO Skin Closure System for its aesthetic outcome and decreased pain [3]. DERMABOND PRINEO Skin Closure System has also led to more cosmetically favorable results assessed via the Hollander Cosmesis Scale and Vancouver Scar Scale [3].

DERMABOND PRINEO Skin Closure System may not be appropriate for all surgical procedures. The package insert cautions against usage in areas of moisture and friction. Thus, for the inframammary fold where moisture and friction are present, DERMABOND PRINEO Skin Closure System may not be ideal.

DERMABOND PRINEO Skin Closure System has demonstrated its efficacy in optimizing scar healing. However, caution must be utilized in its usage, namely, in patients with a prior adhesive allergy and in sites with moisture or friction.

## Figures and Tables

**Table 1:** Patient demographics.

	Patient 1	Patient 2	Patient 3
Age	33	31	38
Race	White	White	African American
BMI	30.5	24.4	28.13
Allergy	Penicillin	NDKA	Codeine, iodine
Rash presented on day	POD 6	POD 28	POD 8
Rash treated with	Medrol, Atarax	Prednisone	Methylprednisolone
Rash resolved on day	POD 19	POD 45	POD 20
